# High Diversity of Myocyanophage in Various Aquatic Environments Revealed by High-Throughput Sequencing of Major Capsid Protein Gene With a New Set of Primers

**DOI:** 10.3389/fmicb.2018.00887

**Published:** 2018-05-03

**Authors:** Weiguo Hou, Shang Wang, Brandon R. Briggs, Gaoyuan Li, Wei Xie, Hailiang Dong

**Affiliations:** ^1^State Key Laboratory of Biogeology and Environmental Geology, China University of Geosciences, Beijing, China; ^2^CAS Key Laboratory of Environmental Biotechnology, Research Center for Eco-Environmental Sciences, Chinese Academy of Sciences (CAS), Beijing, China; ^3^Department of Biological Sciences, University of Alaska Anchorage, Anchorage, AK, United States; ^4^State Key Laboratory of Marine Geology, Tongji University, Shanghai, China; ^5^Department of Geology and Environmental Earth Science, Miami University, Oxford, OH, United States

**Keywords:** myocyanophage, MCP gene, primer, diversity, high-throughput sequencing

## Abstract

Myocyanophages, a group of viruses infecting cyanobacteria, are abundant and play important roles in elemental cycling. Here we investigated the particle-associated viral communities retained on 0.2 μm filters and in sediment samples (representing ancient cyanophage communities) from four ocean and three lake locations, using high-throughput sequencing and a newly designed primer pair targeting a gene fragment (∼145-bp in length) encoding the cyanophage gp23 major capsid protein (MCP). Diverse viral communities were detected in all samples. The fragments of 142-, 145-, and 148-bp in length were most abundant in the amplicons, and most sequences (>92%) belonged to cyanophages. Additionally, different sequencing depths resulted in different diversity estimates of the viral community. Operational taxonomic units obtained from deep sequencing of the MCP gene covered the majority of those obtained from shallow sequencing, suggesting that deep sequencing exhibited a more complete picture of cyanophage community than shallow sequencing. Our results also revealed a wide geographic distribution of marine myocyanophages, i.e., higher dissimilarities of the myocyanophage communities corresponded with the larger distances between the sampling sites. Collectively, this study suggests that the newly designed primer pair can be effectively used to study the community and diversity of myocyanophage from different environments, and the high-throughput sequencing represents a good method to understand viral diversity.

## Introduction

*T4virus* is a genus of viruses that belongs to the order Caudovirales, the family Myoviridae, and the subfamily Tevenvirinae. T4 phages are the archetype of this genus, which efficiently infects *Escherichia coli*. Within this genus are also myocyanophages that infect cyanobacteria and have attracted special attention for their critical role in regulating cyanobacterial population structure, mortality, and evolution (Clokie and Mann, 2006). Myocyanophages are also called T4-like cyanophages for their morphological resemblance to T4 phages. However, phylogenetic analyses based on DNA or amino acid sequences revealed that myocyanophages are distinct from T4 phages or other T4-like viruses (Hambly et al., 2001; Filee et al., 2005; Sullivan et al., 2010; Ignacio-Espinoza and Sullivan, 2012).

Ecologically, myocyanophages are highly diverse and abundant in open-ocean, coastal and estuarine environments, fresh water lakes, and paddy soils (Filee et al., 2005; Comeau and Krisch, 2008; Hellweger, 2009; Sullivan et al., 2010; Wang K. et al., 2011; Wang et al., 2016; Chow and Fuhrman, 2012; Ignacio-Espinoza and Sullivan, 2012; Marston et al., 2013). These viruses play a key role in geochemical cycling of carbon and other elements through their interactions with host bacteria. For example, infection by cyanophages inhibits CO_2_ fixation in cyanobacteria (Bailey et al., 2004; Puxty et al., 2016); viral lysis of cyanobacteria releases cell contents to the organic carbon pool and accelerates carbon cycling in aquatic ecosystems (Weitz and Wilhelm, 2012). However, the photosynthetic genes (*PsbA, PsbD*) present in cyanophages can partially compensate for the loss of the carbon fixation ability of cyanobacteria and protect their hosts against photo-inhibition (Bailey et al., 2004; Hellweger, 2009; Rohwer and Thurber, 2009). Viruses also drive evolution of bacteria by their antagonistic interactions or by introducing new genetic information to bacteria (Lindell et al., 2004; Suttle, 2007; Ignacio-Espinoza and Sullivan, 2012; Marston et al., 2012).

Research in myocyanophage diversity will facilitate a better understanding of their ecological importance. Marker gene and metagenomic analyses are some of the major methods to reveal the viral diversity in various environments (Chow and Fuhrman, 2012; Mizuno et al., 2013; Ma et al., 2014; Labonté et al., 2015). Variable sequence regions (∼400 to ∼800 bp) within those genes encoding g23 major capsid protein (MCP), g20 portal protein, and g43 DNA polymerase gene, have been used in studies of environmental myocyanophage or other *T4virus* diversity (Mann, 2003; Filee et al., 2005; Dekel-Bird et al., 2013; Marston et al., 2013; Needham et al., 2013). However, comparatively long lengths of these genes are not appropriate for sedimentary DNA-based diversity study (ancient DNA) (Boere et al., 2011).

**FIGURE 1:**
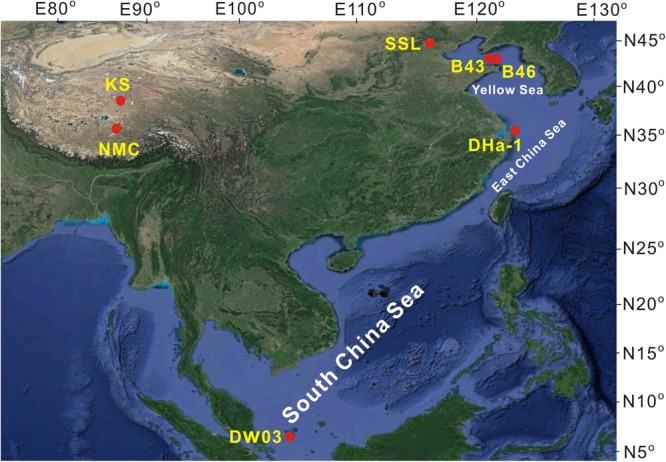
Locations of water and sediment samples used in this study.

Ancient DNA obtained from sediments represents an important source of information on past biodiversity (Pedersen et al., 2015). Short, but variable, DNA markers (<200 bp) were recommended to study past biodiversity (Sønstebø et al., 2010). For example, 60–84-bp length of mammal gene fragments and 10–146 bp length of plant gene fragments were amplified from sediment DNA to reconstruct the livestock farming and landscape histories, respectively (Giguet-Covex et al., 2014). In order to investigate evolution of myocyanophage communities from ancient DNA, we designed a pair of degenerate PCR primers targeting the MCP gene fragment with comparatively short amplicons (∼145-bp). The primer pair was tested with a series of lacustrine and marine samples (**Table 1** and **Figure 1**). In order to assess the effects of sequencing depth on viral diversity estimates, three sequencing efforts with different depths, including clone library sequencing, shallow Illumina sequencing, and deep Illumina sequencing, were conducted, which produced about 100, 1,000–2,000, and more than 50,000 reads, respectively. Thus, the aims of this study were to (1) evaluate the efficiency of the newly designed primers in measuring myocyanophage diversity in various aquatic environments (especially a sediment sample to reveal cyanophage diversity from more than 2,000 years ago), (2) assess the effects of sequencing depth of MCP gene on myocyanophage diversity estimates, and (3) compare the different myocyanophage communities from various aquatic environments.

**Table 1:** Sample location and description.

Sample ID	Name or location	Latitude	Longitude	Sample type	Water type	Date collected
NMC	Namco Lake on Tibetan Plateau	30.51°N	90.50°E	Water sample	Oligsaline	October 13, 2013
KS1	Kusai Lake on Tibetan Plateau	35.69°N	93.00°E	Surface sediment	Saline	October 28, 2010
KS2^∗^	Kusai Lake on Tibetan Plateau	35.69°N	93.00°E	3.85 m below sediment surface	Saline	October 28, 2010
SSL	Shisanling Reservoir in Beijing	40.26°N	116.25°E	Surface sediment	Freshwater	October 1, 2012
B43	Yellow Sea	38.10°N	122.00°E	Surface sediment	Seawater	August 12, 2011
B46	Yellow Sea	37.90°N	122.50°E	Surface sediment	Seawater	August 12, 2011
DHa-1	East China Sea	30.50°N	122.50°E	Surface sediment	Seawater	August 14, 2011
DW03	South China Sea	2.95°N	105.84°E	Water sample	Seawater	July 13, 2012

*^∗^The sample was obtained from a sediment core in October, 2010, with a ^*14*^C calibrated age of ∼2,250 years BP. (Hou et al., 2014).*

## Materials and Methods

### Primer Design

Multiple degenerate primer sets were designed using BioEdit v7.0.1 package^1^ to amplify the myocyanophage g23 MCP gene fragments by identifying conserved sequences after aligning 20 gene sequences retrieved from GenBank. These MCP gene sequences are from 17 representative genomes, which are assigned to CLUSTER A and CLUSTER B (Ignacio-Espinoza and Sullivan, 2012), one freshwater myocyanophage genome, one environmental meta-genome, and one uncultured Mediterranean viral clone sequence. Hairpin check and melting temperature assessment were conducted using Oligo Analyzer 3.1^2^. The primers were then used to amplify myocyanophage MCP genes from water and sediment samples described below (**Table 1**). The amplification efficiencies were assessed according to the brightness of the amplicon bands in agar gel after PCR and electrophoresis. The primer set mcp-821F (CTKGCDGARATYAACMGIGAART) and mcp-966R (ADDAGWCCYTTGAAYTTYTCAAC) targeting partial MCP gene was the most efficient set to amplify the gene (Supplementary Figure S1). On the MCP gene of *Synechococcus* cyanophage S-PM2, primer set mcp-821F-mcp-966R embraces the position from 748 to 893.

### Sample Collection

Surface sediment and water samples were collected from diverse aquatic environments (**Figure 1** and **Table 1**). The list included three lake samples from Tibetan Plateau (one water sample from NMC, two sediments from a KS sediment core, i.e., KS1 and KS2), one lake sediment from Beijing (SSL), two marine sediments from Yellow Sea (B43 and B46), one marine sediment from East China Sea (DHa-1), and one water sample from South China Sea (DW03). Surface sediment samples were collected with a gravity sediment sampler. Planktonic microorganisms and suspension particles were collected onto 0.2 μm membrane filters (Supor-200, Pall Life Sciences) by filtering through about 200 mL water. The above samples were used to analyze viral community (**Table 1**).

### DNA Extraction, Sequencing Strategy

DNA from biomass-containing filters and sediment samples were extracted with modified methods by using MP Biomedical Fast DNA Spin kits (Hou et al., 2013). The polymerase chain reaction (PCR) mix contained 10 ng of template DNA, 2.5 μL rTaq reaction buffer, 400 nM of each primer, 200 mM dNTPs, and 0.3 unit of rTaq polymerase (Takara, Dalian, China) in a 25 μL reaction system. The amplification procedure was as follows: an initial denaturation step at 95°C for 5 min, and 30 cycles of denaturing at 94°C for 30 s, annealing at 54°C for 30 s, and extension at 72°C for 30 s, followed by a final extension step at 72°C for 5 min. PCR products were purified with the Qiagen gel extraction kit (Qiagen, United States) and quantified on a QuBit 2.0 fluorometer (Invitrogen Corp., United States).

Major capsid protein fragments were sequenced with three strategies, i.e., clone-library Sanger sequencing (about 100 reads), shallow Illumina sequencing (1,000–2,000 reads), and deep Illumina sequencing (more than 100,000 reads). Clone sequencing was conducted by cloning the PCR products into pGEM^®^-T Easy Vector Systems (Promega Corporation). One hundred clones for each sample were sequenced with a BigDye Terminator v3.1 sequencing kit (Thermo Fisher Scientific Corporation). In order to sequence the MCP gene on Illumina Miseq platform, Illumina adapter, primer pad, linker, and barcodes were added to the 5′ ends of primer pairs according to a previous publication (Caporaso et al., 2012). Equal amounts of PCR products from all samples were added to the sequencing pool, and the amounts for the shallow Illumina sequencing were reduced by 100 times relative to those used for deep Illumina sequencing. Deep and shallow Illumina sequencing experiments were both conducted with a Miseq V2 (300 cycles) kit by extending 200 cycles, with one primer consisting of the reverse complemented sequence of 3′ Illumina adapter, reverse primer pad, and reverse primer linker, i.e., “CAAGCAGAAGACGGCATACGAGAT AGTCAGCCAG CC” (Caporaso et al., 2012). Using this method, the sequencing reads covered the MCP gene fragments, the forward primer, forward primer linker, forward primer pad, barcode, and 5′ Illumina adapter.

In the case of shallow Illumina sequencing strategy, the MCP gene was sequenced with a thousand sequences on a MiSeq platform. The MCP gene sequences obtained with clone sequencing were deposited in GenBank with accession numbers MG267122 – MG267315. High-throughput sequencing data could be found under a BioProject No. PRJNA393230.

### Data Analysis

Illumina-sequencing errors were minimized by removing reads with low quality scores (<25). Cutadapt 1.10 was used to demultiplex raw sequences and to remove clone-vector and adapter sequences (Matin, 2011). CLC Sequence Viewer 7 (CLC Bio Qiagen, Aarhus, Denmark) was used to translate DNA sequences into amino acid sequences, which was then used to verify the MCP gene. The following steps, including OTU assignment, alpha diversity, and beta diversity calculations, were subsequently completed using the QIIME software package (Caporaso et al., 2010). OTUs were defined at the identity levels of 80, 90, 95, and 97% using UCLUST (Edgar, 2010) based on nucleotide sequences. The first sequence within each OTU cluster was picked as a representative sequence. The representative sequences were aligned with MUSCLE and used to construct a tree using FastTree (Edgar, 2004). Representative sequences were checked by translating into amino acid sequences with CLC Sequence Viewer 7, and further confirmed by protein annotation with NCBI’s Conserved Domain Database (Marchler-Bauer et al., 2017). An amino acid sequence-based maximum-likelihood tree was constructed to display the phylogenetic relations between these dominant representative sequences (representing top-50 abundant OTUs) and reference sequences retrieved from NCBI database using the Jones–Taylor–Thornton evolutionary substitute model (Jones et al., 1992).

Rarefaction curves, used to estimate the myocyanophage richness, were generated with QIIME. The Good’s coverage was used to evaluate sequencing completeness by calculating the probability that a given sequence was chosen from a library of sequencing reads (Good, 1953). Paired sample *T*-test was conducted by using IBM SPSS Statistics 19 to reveal any difference in sequencing Good’s coverage between different sequencing methods. Principal coordinate analyses (PCoAs) were performed to assess the degree of variation of myocyanophage community based on both weighted and unweighted UniFrac distances (Lozupone and Knight, 2005) by using the QIIME software package.

## Results

### Sequencing Profiles

With the newly designed primer, MCP gene was successfully amplified with water (i.e., suspended particles in water obtained by going through 0.2 μm filters) and sediment samples. A total of 746 clone sequences were obtained for eight samples, with an average of 93 ± 4 sequences per sample (Supplementary Table S1). The overall quality scores of deep Illumina sequencing were higher than 25, with an average quality score of 30.8 (Supplementary Figure S2). By filtering out those reads with low quality scores (<25), average number of sequences per sample was 1,471 ± 403 and 144,254 ± 31,878 obtained by shallow, and deep Illumina sequencing, respectively.

### Length of MCP Gene Fragment Amplicons

**FIGURE 2:**
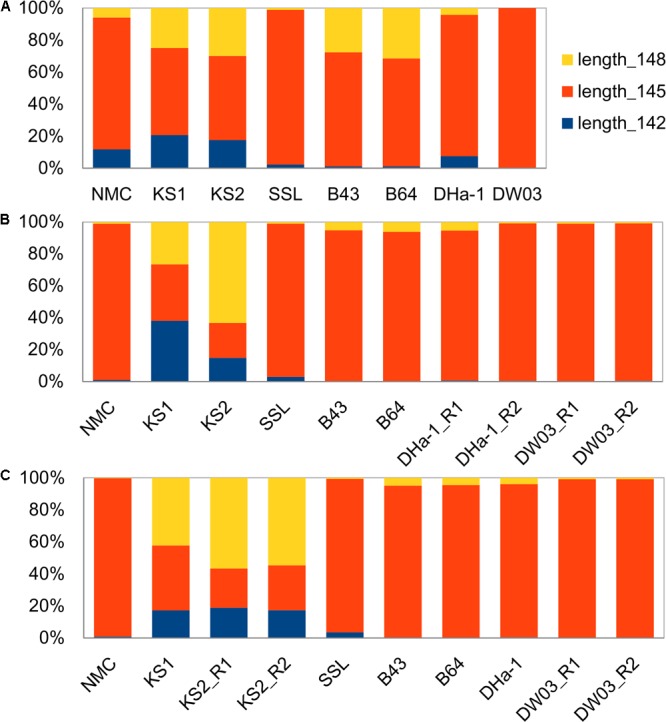
Amplicon length distributions of MCP gene fragments from various samples with clone **(A)**, shallow Illumina sequencing **(B)**, and deep Illumina sequencing **(C)** methods. “R1” and “R2” for some samples refer to two sequencing replicates with different barcodes. Three bar-graphs share the same legends. The Y-axis is relative proportion of each length fragment.

The deep Illumina sequencing reads were variable in length, ranging from < 136-bp to > 154-bp (Supplementary Figure S3). The MCP gene encodes a protein, thus the reads with lengths that did not have a complete codon (i.e., 145 ± 3 *n*, where *n* is any integer) or contained stop codons were discarded. The discarded sequences accounted for an average of 4.6% of total reads in each sample. Overall, the sequences with lengths of 142-, 145-, and 148-bp dominated all samples (97.2–100% after removing non-protein coding sequences; Supplementary Figure S3). However, different samples exhibited different distribution patterns of these three major fragments (**Figure 2**). In two lacustrine (NMC and SSL) and four oceanic (B43, B64, DHa-1, and DW03) samples, 145-bp fragment was predominant (94.8–98.9% in relative percentages, with an average of 97.0% ± 1.8%). In the KS samples, the 148-bp length was most abundant (41.2–56.7% in relative percentages), followed by 145-bp (around 30.1%) and 142-bp (around 17.6%). Thus, the subsequent diversity and community analyses were carried out for these three lengths.

The length distribution of amplified MCP gene fragments based on clone sequencing (**Figure 2A**) was slightly different from those obtained from deep and shallow Illumina sequencing (**Figures 2B,C**). The relative abundance of the 148-bp fragment for B43 and B64 obtained with clone sequencing was higher than that from deep Illumina sequencing. Likewise, for sample NMC, the relative abundances of the 142- and 148-bp fragments from clone sequencing were higher than those from deep Illumina sequencing (**Figure 2**). In sample KS2, the 148-bp fragment was more abundant than the other two. The length distribution of amplified MCP gene fragments based on shallow Illumina sequencing method (**Figure 2B**) was slightly different from both deep Illumina sequencing and clone library methods in that the 148-bp fragment was predominant in the two KS samples. In the other samples, the 145-bp fragment was by far predominant.

Nucleotide and predicted amino acid sequences were identified by comparing them to reference sequences retrieved from GenBank using BlastN and BlastP, and further confirmed by functional protein annotation through NCBI’s Conserved Domain Database (Marchler-Bauer et al., 2017). The results showed that cyanophage MCP gene fragments were predominant (>92%) in the top-200 hit list with a length of 145-bp. Some 142-bp, 148-bp fragments were related to non-cyanophages, including *Sinorhizobium* phages phiN3 and phiM12, *Pseudomonas* phage pf16, *Acidovorax* phage ACP17, and *Ralstonia* phage RSP15, a freshwater phage from Baikal Lake, which is related to *Polynucleobacter* (Cabello-Yeves et al., 2017).

**FIGURE 3:**
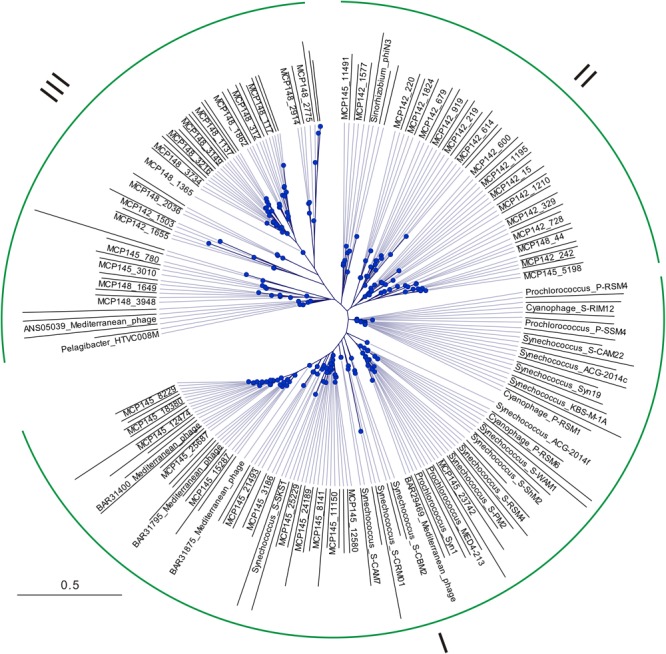
Phylogenetic tree generated with the Maximum-Likelihood method from amino acid sequences derived from partial MCP gene sequences. The tree included representative sequences of top-50 abundant OTUs of 142-, 145-, and 148-bp lengths, as well as their relative sequences retrieved from GenBank. Groups I, II, and III approximately divided by gene fragment lengths 145-, 148-, and 142-bp, respectively. The scale bar refers to evolutionary distance inferred by the Jones–Taylor–Thorn algorithm.

Maximum likelihood phylogenetic tree based on amino acid sequences (**Figure 3**) roughly supported the length-depended lineages with the sequences divided into three major groups. The sequences with 145-bp nucleotide length, mainly distributed in several deep clades (Group I in **Figure 3**), shared close relationships with cyanophage MCP gene fragments and uncultured Mediterranean phage sequences. The majority of 142-bp sequences were divided into two clusters, within Group II (**Figure 3**). In this group, some 142-bp sequences and 145-bp sequences were closely related to *Sinorhizobium* phages. The majority of 148-bp sequences formed Group III (**Figure 3**). In this group, some sequences were closely related to *Pelagibacter* phages and uncultured Mediterranean phages. Other 148-bp sequences (on top of **Figure 3**) were closely related to *Pseudomonas* phage and *Acidovorax* phage.

### Alpha Diversity of MCP Gene Fragment

Operational taxonomic units (OTUs) from all samples were obtained with UCULST method at 80, 90, 95, and 97% similarity levels with deep Illumina sequencing data (Supplementary Figure S4). Around 1,000, 4,000, 20,000, and 45,000 OTUs were obtained at four similarity levels, respectively. For the tractability of OTU numbers, the OTU table at similarity 90% was chosen hereafter. At 90% similarity, the highest numbers of OTUs after removing singletons were observed in Numco Lake water sample and the South China Sea water sample DW03 in the deep Illumina sequences (Supplementary Figure S5).

**FIGURE 4:**
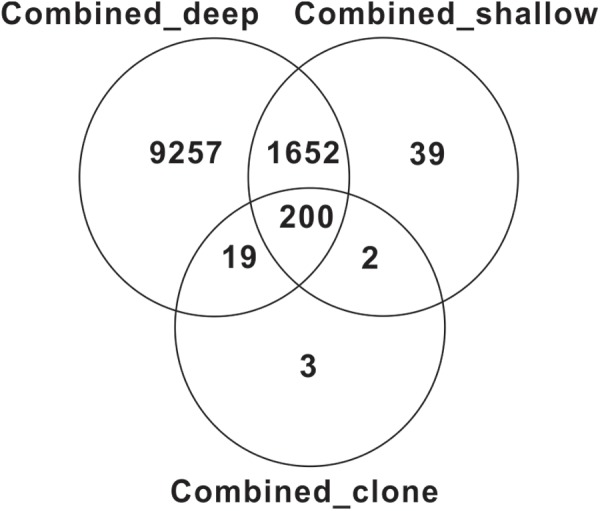
Venn diagram showing common OTUs obtained by three sequencing methods.

**FIGURE 5:**
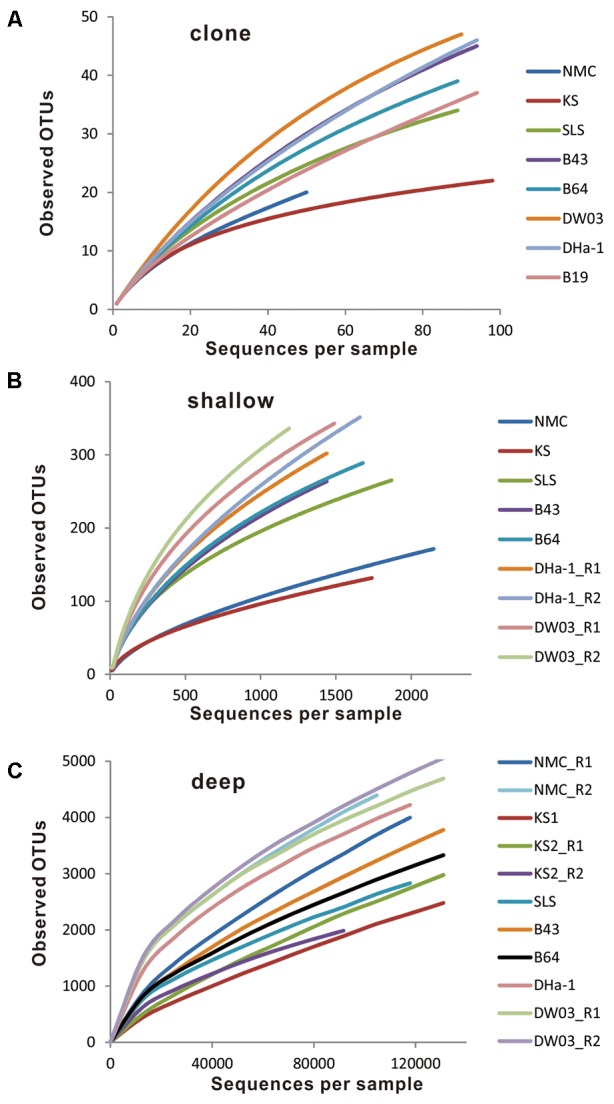
Rarefaction curves by plotting observed OTU number against sampled sequence number. **(A)** Clone sequence, **(B)** shallow Illumina sequence, and **(C)** deep Illumina sequence.

Venn diagrams showed that most OTUs from the deep sequencing effort were also captured by the shallow sequencing (**Figure 4**). For example, most OTUs from a combined clone library for all samples were detected by both shallow and deep Illumina sequencing methods, and most OTUs from shallow Illumina sequencing were also detected by deep Illumina sequencing (**Figure 4**). Similar overlapping pattern also occurred at the individual sample level (Supplementary Figure S6). However, rarefaction curves did not reach plateaus for all samples with all three sequencing methods (**Figure 5**). As expected, the coverage value for full-length MCP gene fragments was dependent on sequencing depth, i.e., deeper sequencing resulted in a higher coverage (**Table 2**).

**Table 2:** Good’s coverage values for the samples with different sequencing depths and lengths.

Sample ID	Clone sequencing with 50 sub-sampling depth	Shallow sequencing with 1,600 sub-sampling depth	Deep sequencing with 38,000 sub-sampling depth	Deep sequencing with 130,000 sub-sampling depth
NMC	0.740	0.944	0.968	0.976
KS1	0.910	0.980	0.981	0.986
KS2	0.918	0.953	0.979	0.985
SSL	0.811	0.926	0.978	0.985
B43	0.716	0.900	0.972	0.980
B64	0.737	0.916	0.970	0.984
DHa-1	0.684	0.875	0.968	0.981
DW03	0.744	0.865	0.966	0.982

### Beta Diversity

Principal coordinate analysis based on deep sequencing results revealed that, within the marine samples, the communities displayed significant variations. As the distance between the samples increased, myocyanophage community became increasingly dissimilar (Supplementary Figure S7). However, such effect was not observed for the lake samples (data not shown).

**FIGURE 6:**
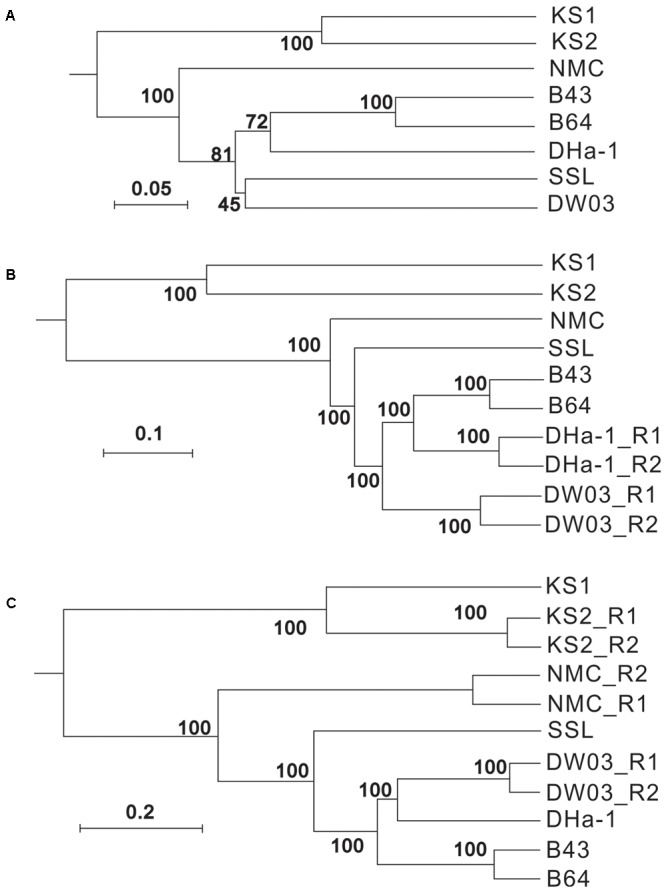
UPGMA cluster trees based on unweighted UniFrac distances. The numbers on the nodes refer to the support percentages by 1,000 jackknife tests. UPGMA cluster trees in **(A–C)** were constructed based on clone sequences, shallow Illumina sequences, and deep Illumina sequences, respectively. “_R1” and “_R2” in sample ID refer to two sequencing replicates with different barcodes.

**FIGURE 7:**
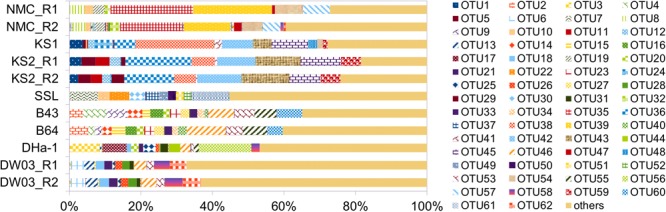
Distribution of dominant, full-length MCP gene OTUs (relative abundances higher than 2%) in eight samples. These sequences were obtained based on deep Illumina sequencing.

Different sequencing methods yielded similar myocyanophage community groupings across different samples (**Figure 6**). The KS samples were the most different from others and they formed a separate cluster, likely because of higher abundances of 142- and 148-bp sequences relative to other samples. The composition of dominant OTUs (relative abundances >2%), based on deep Illumina sequencing, was different across different samples (**Figure 7**). Specifically, oceanic (B43, B64, and DHa-1, and DW03) and lacustrine samples did not share any dominant OTUs (0/65). One common OTU (1/30) was observed across all marine samples, 11 OTUs (11/30) were common in two Yellow Sea samples (B43 and B64), and 2 OTUs (2/17) were common to DHa-1 and DW03. Dominant OTUs were not shared between any lakes. Within the same lake (e.g., KS), 6 dominant OTUs (6/14) were shared between the two KS sediment samples.

## Discussion

### Sensitivity and Specificity the Newly Designed Primer

In this study, a MCP gene fragment was successfully amplified from all studied aquatic samples (DNA extracted from suspended particles retained on filters and sediment samples) by using a newly designed primer. A large range of the amplicon length (from 136-bp to 154-bp) suggested some non-specific amplification. However, other than the Kusai Lake samples, the dominance of 145-bp amplicon length (higher than 92.0%, represented myocyanophage) in most samples suggested a high specificity of the newly designed primer set. In the Kusai Lake samples, three amplicon lengths, i.e., 142-, 145-, and 148-bp, all occurred and the 142- and 148-bp amplicons represented non-cyanophages. The difference in the viral communities between Kusai Lake and other aquatic environments may be caused by the presence of both myocyanophages and non-myocyanophages in the Kusai Lake, whereas other lakes or oceans were dominated by myocyanophages. According to the extent of degeneration, the primer set designed in this study may capture more myocyanophages than MZIA1bis-MZIA6 primer set (Filee et al., 2005), but less myocyanophages than T4superF1-T3superR1 primer set (Chow and Fuhrman, 2012).

Comparatively, a high percentage of non-myocyanophage was detected in lacustrine samples, especially for the Kusai Lake samples. The hosts of some non-cyanophages appear to be soil bacteria. For example, species of *Sinorhizobium* are soil bacteria capable of nodulating leguminous plants (Johnson et al., 2015); species of *Ralstonia* are soil phyto-pathogens (Fujiwara et al., 2008). Thus, these viruses with amplicon lengths of 142- and 148-bp may represent terrestrial input to lakes, and it is reasonable to observe that the lake samples contained higher percentage of 142- and 148-bp MCP fragments than the oceanic samples, because the lakes in this study have higher terrestrial input than the oceans.

### Sample Types in This Study

In this study, sediment particles and microbial cells retained on 0.2 μm membrane filters were used in viral community analyses, which is different from previous studies using ultracentrifugation, tangential flow filtration, or ultrafiltration (Chow and Fuhrman, 2012; Chenard et al., 2015; Cai et al., 2016). Therefore, the viral communities determined in this study should represent bacteria and sediment-attached particle rather than the free-living viral particles.

Another sample type used in this study was sediment. Myocyanophage MCP genes detected in these sediment samples should have been derived from water column but settled into sediments. Indeed, our previous study detected cyanobacteria genes in Kusai Lake sediment (Hou et al., 2014). A similar observation was also made in the subtropical Pearl River (He et al., 2017), where cyanophage sequences were detected in estuarine sediments.

### Myocyanophage Diversity Estimate in Various Environments

T4-like phages, including cyanophages, belong to a superfamily (Filee et al., 2005; Comeau and Krisch, 2008), and are widespread in various environments (Comeau and Krisch, 2008). The MCP gene is one of the quickly evolving genes involved in the interaction between viruses and their hosts (Marston et al., 2012). As a result, it is not surprising that extremely diverse viruses were detected from various water and sediment samples. Myocyanophage communities have been extensively studied based on diversity surveys of MCP gene using various primers such as MZIA1bis and MZIA6 (Filee et al., 2005) or T4superF1 and T3superR1 (Chow and Fuhrman, 2012), which amplify the similar region of MCP gene fragments, from position ∼300 to ∼760 on *Synechococcus* cyanophage S-PM2. These primer pairs amplify the MCP gene fragments of ∼400–∼500 bp in length. The short primer pair designed in this study, amplifying a different region, was initially intended to study myocyanophage community variation based on ancient DNA preserved in lake sediments in response to environmental change. A comparison between our results and those from a previous study (Chow and Fuhrman, 2012) revealed that when the sequencing depth is similar and percent identity is at 90%, shorter sequences (e.g., 145-bp obtained in this study) resulted in more OTUs than longer sequences (∼400 to ∼500 bp as obtained by Chow and Fuhrman, 2012), which may be caused by location of the 145-bp amplicon in a more variable region of the MCP gene.

### Variations in Myocyanophage Community

Overall, the significant variations of myocyanophage community from different samples observed in this study may be collectively caused by different distributions of host cyanobacteria in different environmental settings. Phylogenetic analysis suggested that cyanobacteria from Tibetan lakes formed different clusters, separated from other habitats, including other freshwater lakes and marine samples (Wu et al., 2010), which may be the fundamental reason for different myocyanophage community in the different environments (**Figures 6, 7**). Wang G. et al. (2011) also attributed their observed variation of viral community to different distribution of host cyanobacteria. Two previous studies on estuary viral communities identified marine and freshwater viral biomes in the mixing zone (Cai et al., 2016; He et al., 2017) which is also the mixing zone of the marine and freshwater viral hosts. These results collectively suggested that variation of hosts affect the T4-like viral community composition. Although the Kusai Lake and Namuco Lake are both located on the Tibetan Plateau, but the myocyanophage communities were very different, which may be caused by geographic separation of both cyanophages and their hosts. For the marine samples, the circulating ocean current may have led to transport of both microbes and viruses across different oceans. As a result, more common myocyanophage groups or OTUs were found within marine samples, and the communities were more similar if the geographic distances were close (Supplementary Figure S7). Substantial difference in the myocyanophage communities between the surface sediment and deep sediment in the Kusai Lake samples (**Figure 6**) may be caused by temporal variations in hydrological and climate conditions, because these two samples should have been deposited at different times. The viral community in the surface sediment of Kusai Lake represents a modern community, and the community from the 3.85 m depth represents an ancient community from 2,250 years cal. before present (BP) (Hou et al., 2014). According to the result from the deep sequencing effort, the read ratio of 142-/145-bp, an estimate of terrestrial input relative to lacustrine production, was higher for the deeper sample (KS2) than that for the surface sediment sample (KS1), suggesting a higher terrestrial input 2,250 years cal. BP. This result is consistent with our earlier ancient DNA based study (Hou et al., 2014) and supports a strong summer monsoon scenario during that time (Liu et al., 2009). In summary, the newly designed primer was effective in studying the diversity of myocyanophages in various aquatic samples, including sediment samples representing cyanophage diversity more than 2,000 years ago. Due to high diversity, deep sequencing presents a good method to understand myocyanophage community. The amplicons were mainly composed of 142-, 145-, and 148-bp fragments. These different lengths of the gene fragments may represent source variations, terrestrial source or aquatic sources. The myocyanophage communities were also more heterogeneous in lakes than in oceans. Due to connection via oceanic currents, the marine viral communities displayed a distance effect, i.e., with increased geographic distances, the oceanic viral communities became increasingly dissimilar.

## Author Contributions

WH and HD conceived and designed the experiments. WH and SW performed the experiments. WX contributed the sampling vessel and tools. WH, BB, and GL analyzed the data. WH, SW, BB, GL, WX, and HD wrote the paper.

## Conflict of Interest Statement

The authors declare that the research was conducted in the absence of any commercial or financial relationships that could be construed as a potential conflict of interest. The reviewer RZ and the handling Editor declared their shared affiliation.

## Footnotes

1 http://www.mbio.ncsu.edu/BioEdit/bioedit.html

2 http://sg.idtdna.com/calc/analyzer
