# Supplementary material for: High Diversity of Myocyanophage in Various Aquatic Environments Revealed by High-Throughput Sequencing of Major Capsid Protein Gene With a New Set of Primers
Source: Front Microbiol. 2018 May 3;9:887. doi: 10.3389/fmicb.2018.00887 (PMC5943533; doi:10.3389/fmicb.2018.00887)
Supplement: Supplementary file 1 [file Table_1.PDF]

Table S1. Sequencing numbers obtained from different sequencing efforts, and relative percentages of dominant sequence lengths within total sequencing numbers.

Sample ID	numbers of clone sequences	numbers of shallow Illumina sequences	Deep Illumina sequences	
			number	Average percentage of 142-, 145-, and 148-bp fragments
NMC-R1	100	1761	133985	95.2%
NMC-R2	/	/	165576	95.6%
KS1	92	2363	139420	96.3%
KS2-R1	98	1308	158652	94.9%
KS2-R2	/	/	107620	95.7%
SSL	89	1573	135328	95.4%
B43	94	1265	140901	93.1%
B64	89	1507	155493	93.0%
DHa-1	94	1212	136584	95.8%
DW03-R2	90	995	227120	95.4%
DW03-R1	/	1260	201818	94.7%
