# Supplementary material for: High Diversity of Myocyanophage in Various Aquatic Environments Revealed by High-Throughput Sequencing of Major Capsid Protein Gene With a New Set of Primers
Source: Front Microbiol. 2018 May 3;9:887. doi: 10.3389/fmicb.2018.00887 (PMC5943533; doi:10.3389/fmicb.2018.00887)
Supplement: Supplementary file 2 [file Image_1.PDF]

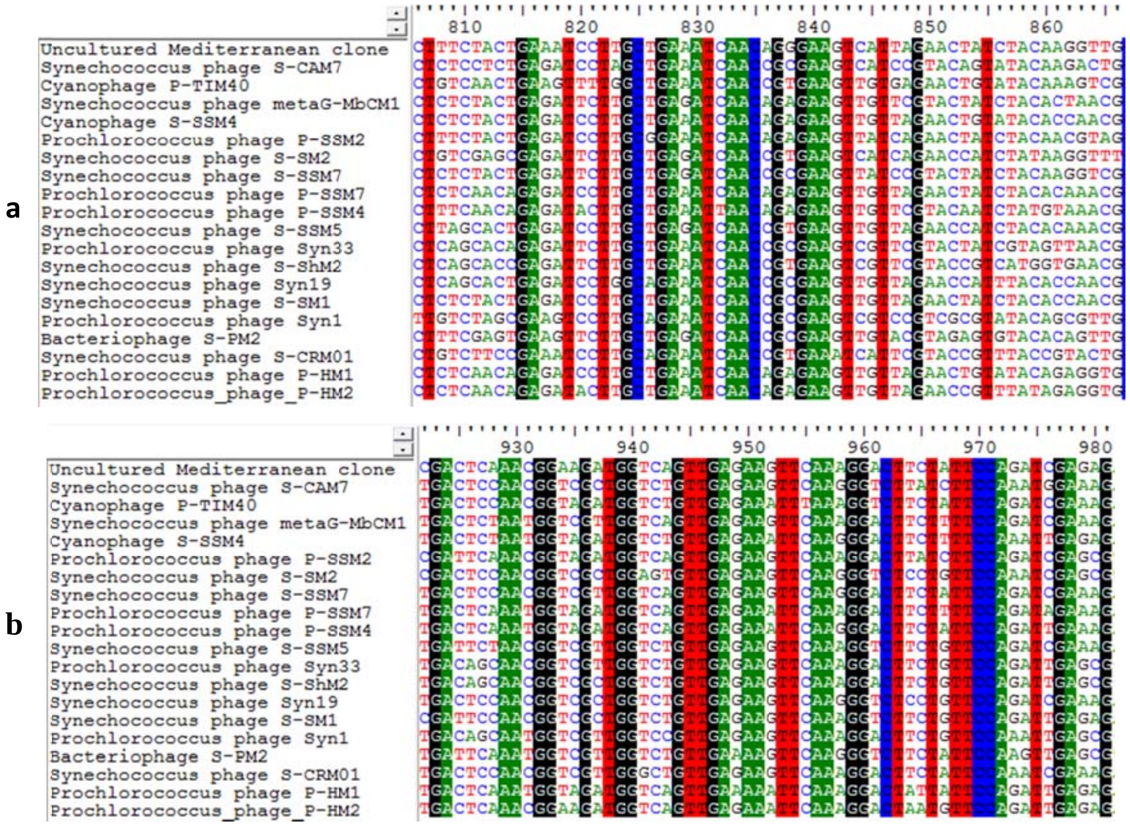

Fig. S1. Primer design by aligning full-length T4viral g23 MCP genes from Genbank using BioEdit v7.0.1. Sequence position 821 – 841 (a) and sequence position 944 – 966 were selected as forward mcp-821F (5’-CTKGCDGARATYAACMGIGAART-3’) and reverse primer mcp-966R (5’-ADDAGWCCYTTGAAYTTYTCAAC-3’), respectively. Heterogeneous positions were replaced with degenerated bases. The accession numbers of those sequences used for primer design included KT997817.1, KU686212.1, KP211958.1, JN371769.1, HQ316583.1, AY939844.2, GU071095.1, GU071098.1, GU071103.1, AY940168.2, GU071097.1, GU071108.1, GU071096.1, GU071106.1, GU071094.1, GU071105.1, AJ630128.1, HQ615693.1, GU071101.1, and GU075905.1.
