# Supplementary material for: High Diversity of Myocyanophage in Various Aquatic Environments Revealed by High-Throughput Sequencing of Major Capsid Protein Gene With a New Set of Primers
Source: Front Microbiol. 2018 May 3;9:887. doi: 10.3389/fmicb.2018.00887 (PMC5943533; doi:10.3389/fmicb.2018.00887)
Supplement: Supplementary file 3 [file Image_2.PDF]

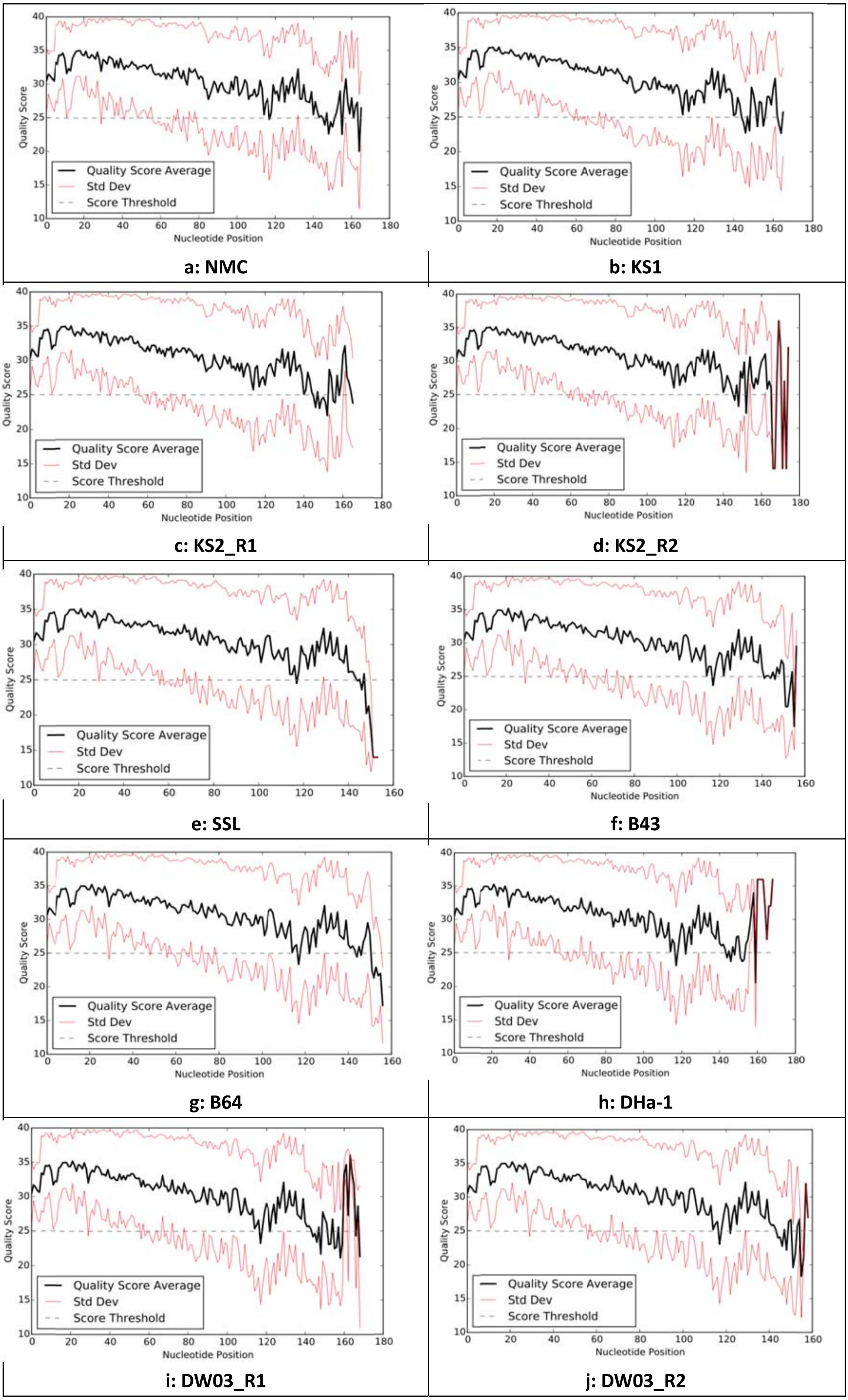

Fig. S2. Quality score plots for individual samples. The overall quality scores were higher than 25. “R1” and “R2” for some samples refer to two sequencing replicates with different barcodes.
