# Supplementary material for: High Diversity of Myocyanophage in Various Aquatic Environments Revealed by High-Throughput Sequencing of Major Capsid Protein Gene With a New Set of Primers
Source: Front Microbiol. 2018 May 3;9:887. doi: 10.3389/fmicb.2018.00887 (PMC5943533; doi:10.3389/fmicb.2018.00887)
Supplement: Supplementary file 4 [file Image_3.PDF]

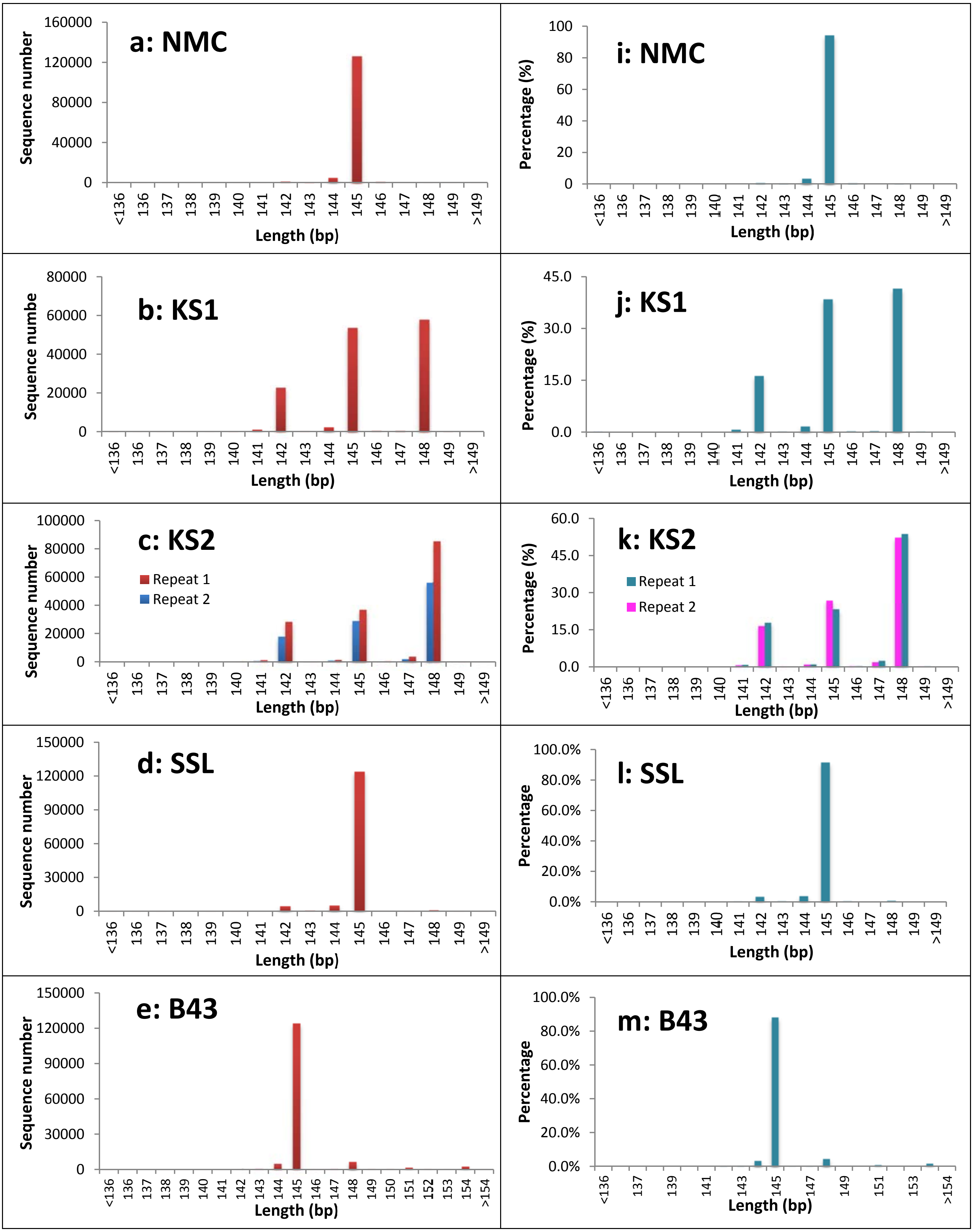
a: NMC
Sequence number
0
40000
80000
120000
160000
Length (bp)
<136 136 137 138 139 140 141 142 143 144 145 146 147 148 149 >149
i: NMC
Percentage (%)
0
20
40
60
80
100
Length (bp)
<136 136 137 138 139 140 141 142 143 144 145 146 147 148 149 >149
b: KS1
Sequence numbe
0
20000
40000
60000
80000
Length (bp)
<136 136 137 138 139 140 141 142 143 144 145 146 147 148 149 >149
j: KS1
Percentage (%)
0.0
15.0
30.0
45.0
Length (bp)
<136 136 137 138 139 140 141 142 143 144 145 146 147 148 149 >149
c: KS2
Repeat 1
Repeat 2
Sequence number
0
20000
40000
60000
80000
100000
Length (bp)
<136 136 137 138 139 140 141 142 143 144 145 146 147 148 149 >149
k: KS2
Repeat 1
Repeat 2
Percentage (%)
0.0
15.0
30.0
45.0
60.0
Length (bp)
<136 136 137 138 139 140 141 142 143 144 145 146 147 148 149 >149
d: SSL
Sequence number
0
30000
60000
90000
120000
150000
Length (bp)
<136 136 137 138 139 140 141 142 143 144 145 146 147 148 149 >149
l: SSL
Percentage
0.0%
20.0%
40.0%
60.0%
80.0%
100.0%
Length (bp)
<136 136 137 138 139 140 141 142 143 144 145 146 147 148 149 >149
e: B43
Sequence number
0
30000
60000
90000
120000
150000
Length (bp)
<136 136 137 138 139 140 141 142 143 144 145 146 147 148 149 150 151 152 153 154 >154
m: B43
Percentage
0.0%
20.0%
40.0%
60.0%
80.0%
100.0%
Length (bp)
<136 137 139 141 143 145 147 149 151 153 >154

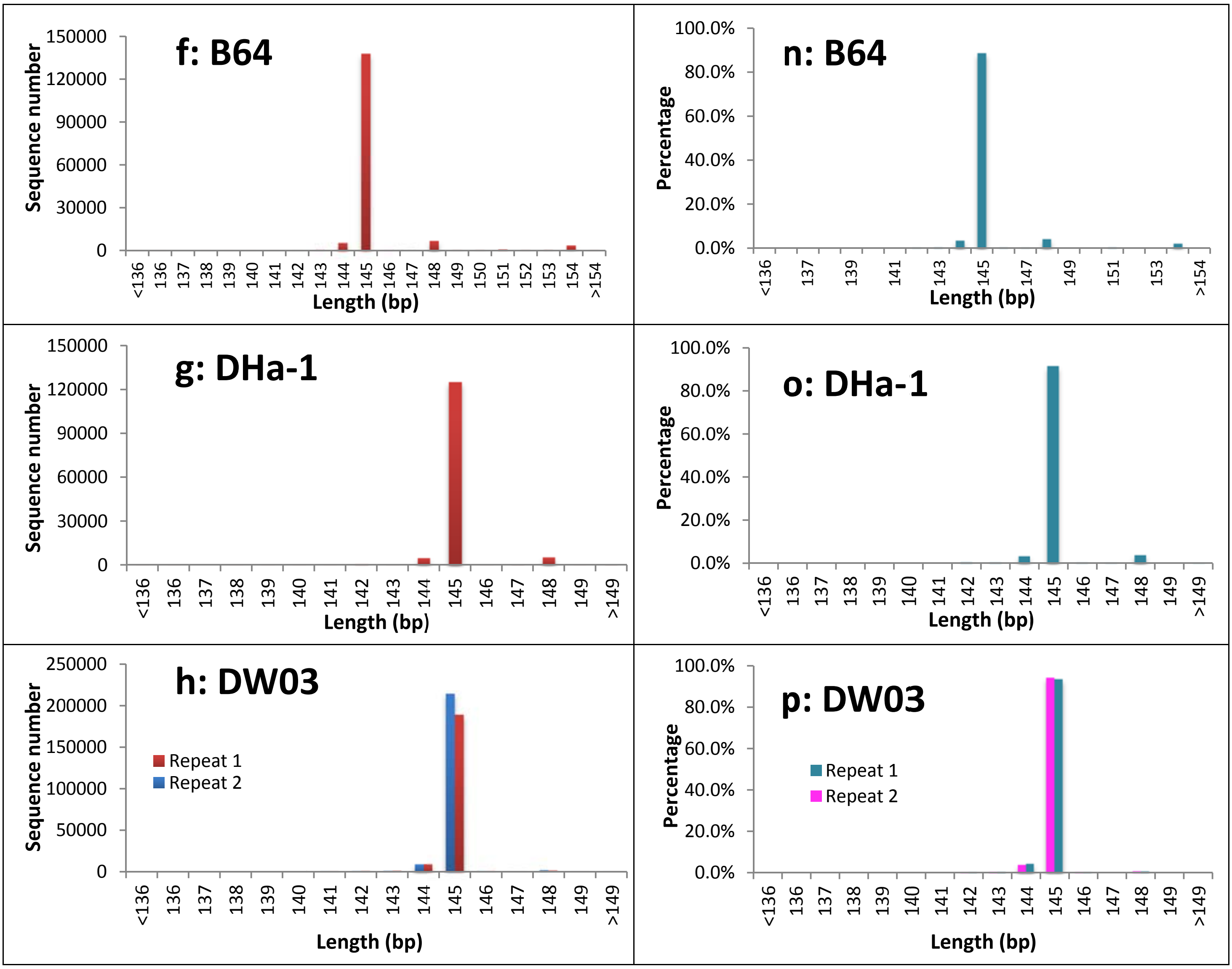

Fig. S3. Sequence numbers (a – h) and relative percentages (i – p) of different length amplicons obtained with deep Illumina sequencing.
