# Supplementary material for: High Diversity of Myocyanophage in Various Aquatic Environments Revealed by High-Throughput Sequencing of Major Capsid Protein Gene With a New Set of Primers
Source: Front Microbiol. 2018 May 3;9:887. doi: 10.3389/fmicb.2018.00887 (PMC5943533; doi:10.3389/fmicb.2018.00887)
Supplement: Supplementary file 5 [file Image_4.PDF]

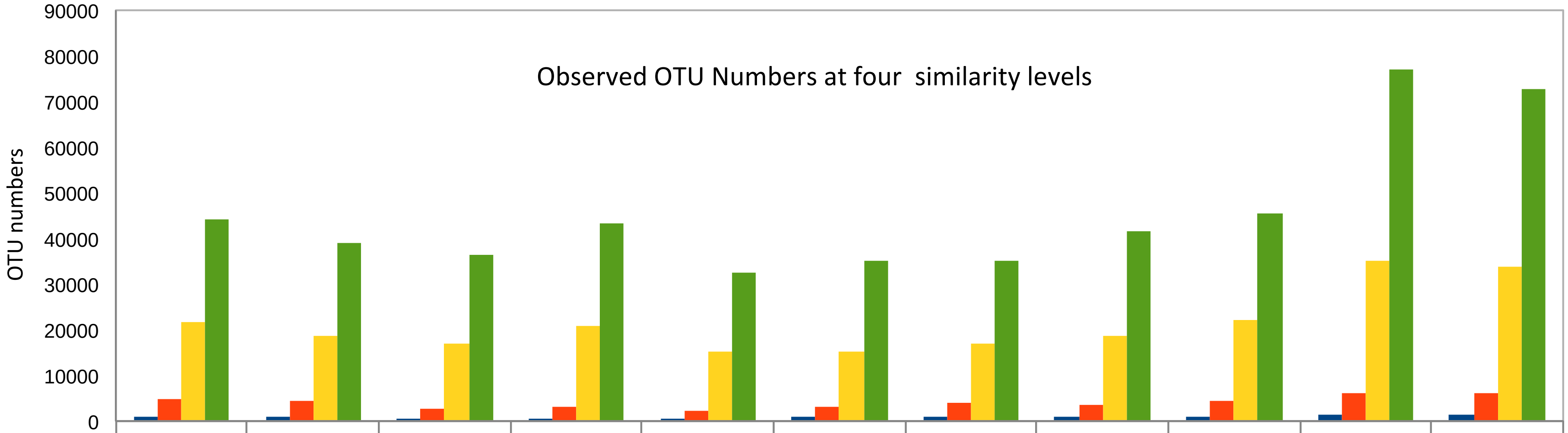

	NMC_R1	NMC_R2	KS1	KS2_R1	KS2_R2	SSL	B43	B64	DHa-1	DW03_R1	DW03_R2
80%	912	891	455	416	281	823	1038	961	1116	1164	1164
90%	4646	4235	2495	3312	2125	3004	3782	3545	4464	6026	6172
95%	21570	18747	17066	20977	15014	15154	17062	18801	22058	35286	33930
97%	44159	38919	36332	43466	32664	35303	35159	41621	45370	76992	72808

Fig. S4. Observed OTU numbers at 80%, 90%, 95%, and 97% similarity levels obtained from deep Illumina sequencing. At 144,254 ± 31,878 sequencing depth, 838, 3982, 21424, and 45708 OTUs were obtained at 80%, 90%, 95%, and 97% similarity levels, respectively.
