# Supplementary material for: High Diversity of Myocyanophage in Various Aquatic Environments Revealed by High-Throughput Sequencing of Major Capsid Protein Gene With a New Set of Primers
Source: Front Microbiol. 2018 May 3;9:887. doi: 10.3389/fmicb.2018.00887 (PMC5943533; doi:10.3389/fmicb.2018.00887)
Supplement: Supplementary file 6 [file Image_5.PDF]

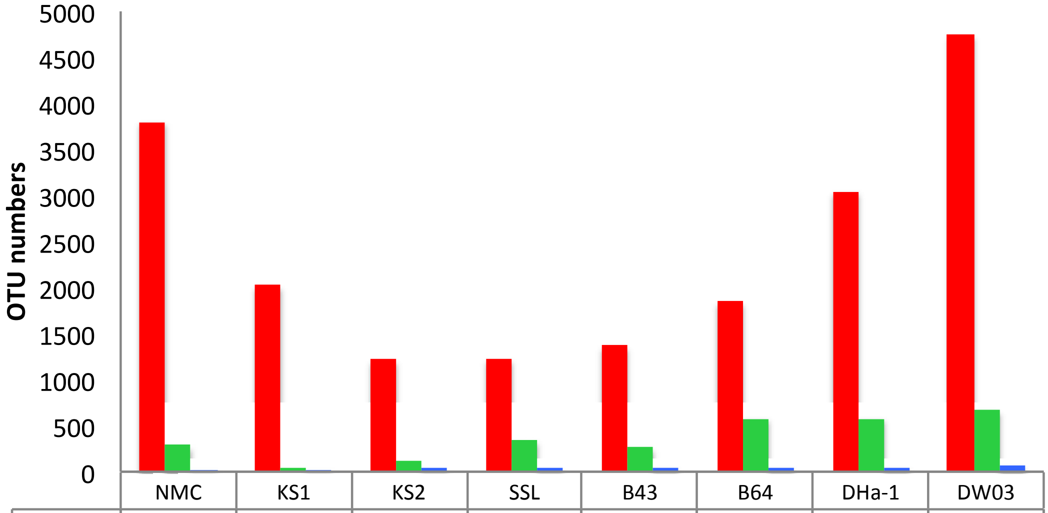

	NMC	KS1	KS2	SSL	B43	B64	DHa-1	DW03
deep	3801	2025	1216	1226	1366	1846	3041	4742
shallow	282	47	117	344	266	583	583	665
clone	24	27	32	41	47	48	48	64

Fig. S5. OTU numbers (after deleting singletons in a combined OTU table) at 90% similarity obtained from deep Illumina sequencing, shallow Illumina sequencing, and clone sequencing, respectively.
