# Supplementary figures and images for: High Diversity of Myocyanophage in Various Aquatic Environments Revealed by High-Throughput Sequencing of Major Capsid Protein Gene With a New Set of Primers
Source: Front Microbiol. 2018 May 3;9:887. doi: 10.3389/fmicb.2018.00887 (PMC5943533; doi:10.3389/fmicb.2018.00887)

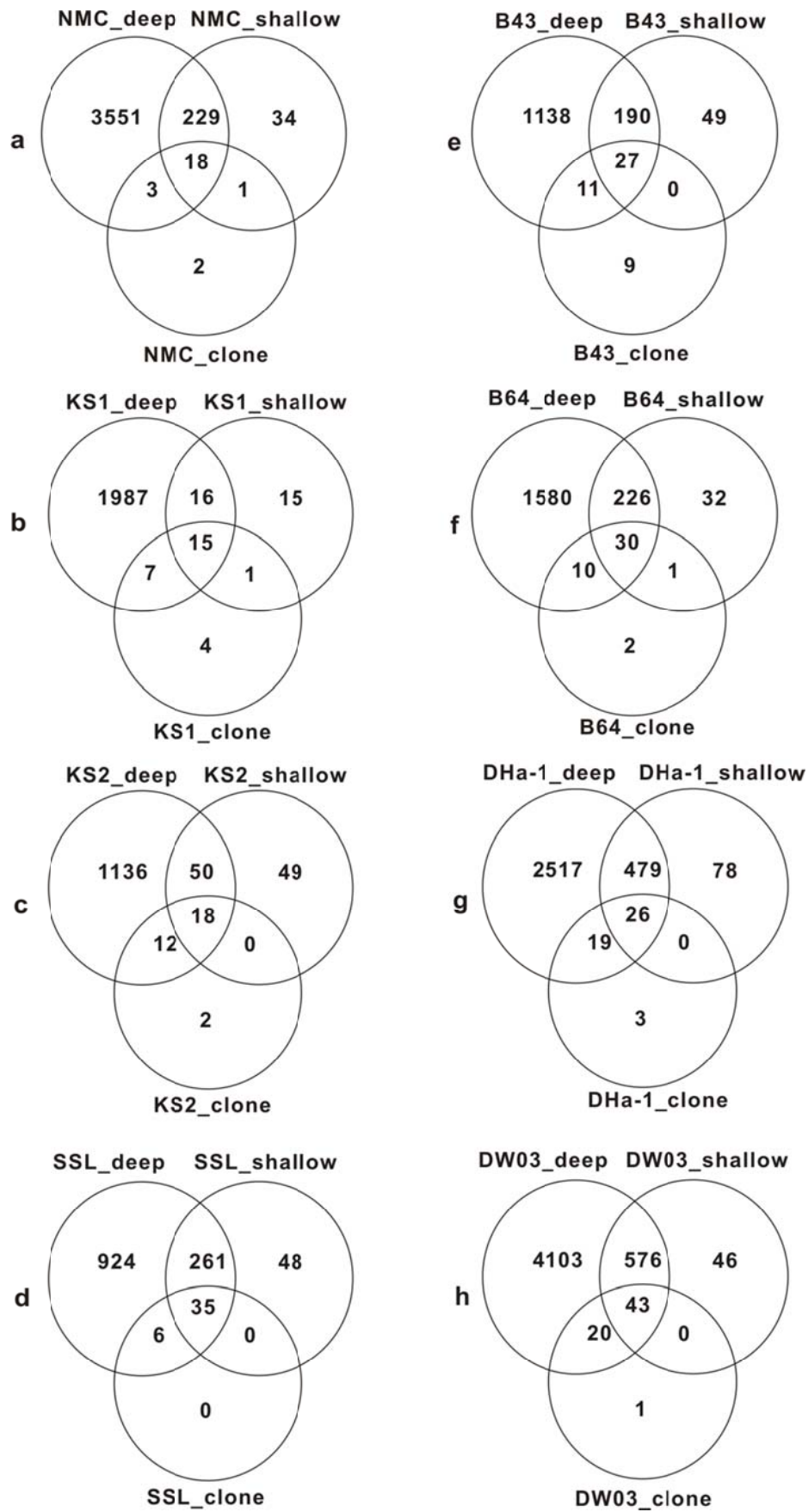

**Fig. S6.** Venn diagram showing sharing of OTUs by three sequencing strategies for every sample

Supplement: Supplementary file 7 [file Image_6.PDF]
