# Supplementary material for: High Diversity of Myocyanophage in Various Aquatic Environments Revealed by High-Throughput Sequencing of Major Capsid Protein Gene With a New Set of Primers
Source: Front Microbiol. 2018 May 3;9:887. doi: 10.3389/fmicb.2018.00887 (PMC5943533; doi:10.3389/fmicb.2018.00887)
Supplement: Supplementary file 8 [file Image_7.PDF]

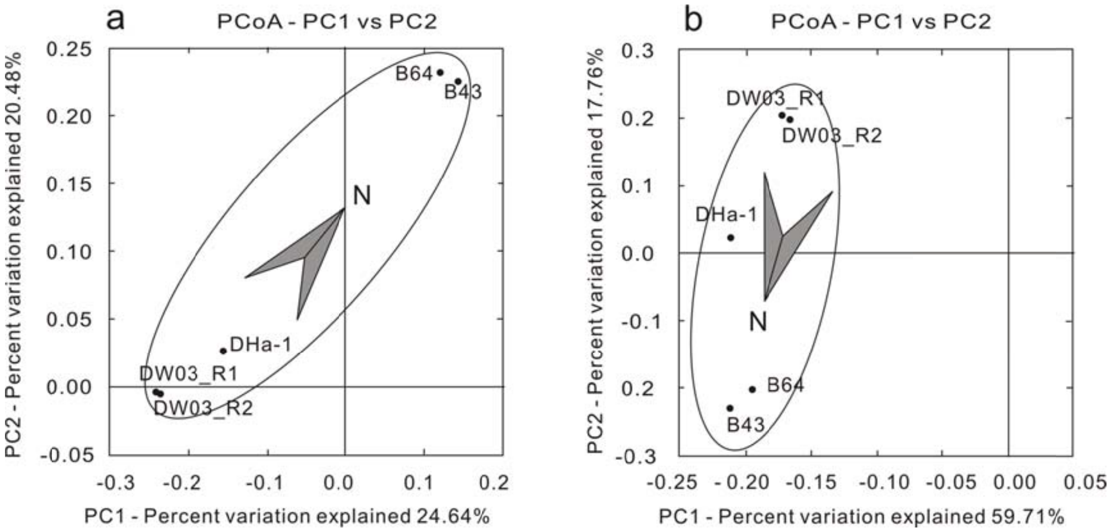

Fig. S7. PCoA plots based on the unweighted UniFrac distance matrix (a) and weighted UniFrac distance matrix (b). "N" refers to approximate south-north direction for the marine samples (Fig. 1).
